# The Effect of Gamma and Beta Radiation on a UVTRON Flame Sensor: Assessment of the Impact on Implementation in a Mixed Radiation Field

**DOI:** 10.3390/s18124394

**Published:** 2018-12-12

**Authors:** Anita J. Crompton, Kelum A. A. Gamage, Divyesh Trivedi, Alex Jenkins

**Affiliations:** 1 Engineering Department, Lancaster University, Lancaster LA1 4YW, UK; 2 School of Engineering, University of Glasgow, Glasgow G12 8QQ, UK; kelum.gamage@glasgow.ac.uk; 3 The National Nuclear Laboratory, Warrington WA3 6AE, UK; divyesh.trivedi@nnl.co.uk; 4 Characterisation, Inspection & Decontamination Group, Sellafield Ltd., Cumbria CA20 1PG, UK; alex.jenkins@sellafieldsites.com

**Keywords:** UVTRON flame detector, alpha detection, alpha-induced radioluminescence, gamma radiation, beta radiation, nuclear decommissioning

## Abstract

Due to the short path length of alpha particles in air, a detector that can be used at a distance from any potential radiological contamination reduces the time and hazard that traditional alpha detection methods incur. This would reduce costs and protect personnel in nuclear power generation and decommissioning activities, where alpha detection is crucial to full characterisation and contamination detection. Stand-off alpha detection could potentially be achieved by the detection of alpha-induced radioluminescence, especially in the ultraviolet C (UVC) wavelength range (180–280 nm) where natural and artificial background lighting is less likely to interfere with detection. However, such a detector would also have to be effective in the field, potentially in the presence of other radiation sources that could mask the UVC signal. This work exposed a UVC sensor, the UVTRON (Hamamatsu, Japan) and associated electronics (driver circuit, microprocessor) to sources of beta and gamma radiation in order to assess its response to both of these types of radiation, as may be found in the field where a mixed radiation environment is likely. It has been found that the UVTRON is affected by both gamma and beta radiation of a magnitude that would mask any UVC signal being detected. ^152^Eu generated 0.01 pulses per second per Bq through beta and gamma interactions, compared to ^210^Po, which generates 4.72 × 10^−8^ cps per Bq from UVC radioluminescence, at 20 mm separation. This work showed that UVTRON itself is more susceptible to this radiation than the associated electronics. The results of this work have implications for the use of the UVTRON as a sensor in a stand-off detection system, highlighting the necessity for shielding from both potential gamma and beta radiation in any detector design.

## 1. Introduction

The short travel of alpha particles in air creates difficulties in the detection of alpha-emitting materials due to the requirement for any directly interacting detector to be around 10 mm from the surface being scanned [1]. In the case of a large surface area or complex geometry, this is time consuming. Where there is a mixed radiation field, this can also pose a potential hazard to detector operators, and may require PPE (Personal Protective Equipment) and limited exposure times. Hence, a stand-off detector is preferable to reduce costs, reduce time and limit any hazard to personnel. As they travel from the emitting source, alpha particles ionise the air, which generates radioluminescence photons, mainly in the ultraviolet wavelength range. These photons travel in the order of kilometres, much further than the alpha particles themselves, which are limited to approximately 50 mm, depending on their energy. This radioluminescence therefore presents an opportunity for stand-off alpha detection for nuclear operation, decommissioning and security applications. Several previous studies have been made of the radioluminescence phenomenon, and possible detector configurations have been put forward [2]. These have mainly focused on the ultraviolet A and B wavelength ranges, 300–400 nm, where most of the radioluminescence is generated. However, there is great deal of background interference within this wavelength range, due to sunlight and emissions from artificial lighting.

The UVTRON flame sensor made by Hamamatsu is designed to detect UVC emissions from flames as part of fire warning systems [3]. It uses this wavelength range as sunlight in this range is stopped by the atmosphere, making the UVTRON what is termed ‘solar-blind’. Artificial lighting also does not emit UVC, as it is harmful to human eyes, and is a waste of energy to generate as it does not aid vision [4]. Therefore, there is little or no background light in this wavelength range to interfere with detection of the flame emissions, especially indoors. When UVC photons are detected by the UVTRON, it outputs a pulse which can be detected directly, or more usually can be processed by an especially designed driver circuit (C10807, Hamamatsu, Japan), also available commercially off-the-shelf (COTS) from Hamamatsu, and a 5 V square wave is emitted, which is transmitted to some configuration of warning device to alert people to the presence of a flame.

In previous work, the ability of the UVTRON to detect the UVC wavelength portion of the UV emission from an alpha emitter, ^210^Po, was established [5]. This introduced the potential of the UVTRON as the sensing element in a stand-off alpha detector. However, as polonium is a pure alpha emitter, this work did not establish the effect of other types of radiation, as may be found in the field, on the UVTRON. Further work has been undertaken to assess the reaction of this sensor to the presence of different gases [6] with a view to determining its potential for use in a stand-off alpha detection system. The work detailed in this paper was carried out to determine the effect of beta and gamma radiation on the UVTRON and its associated electronics in order that the design of a detector system using the UVTRON could take into account and minimise or eradicate the effect of this on the determination of the UVC emissions.

## 2. Materials and Methods

Initial experiments were carried out at the National Nuclear Laboratory (NNL), Cumbria, UK, and subsequent experiments were carried out at Lancaster University, Lancashire, UK. The initial experiments were used to inform the planning of the second set; however, some have been included here where relevant.

**Figure 1:**
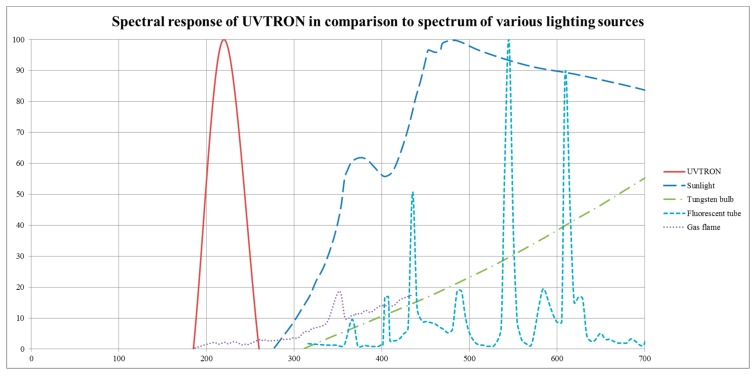
Spectral response of the R9533 UVTRON. Derived from data from Hamamatsu [7] and OSRAM [8].

The UVTRON is a solar-blind flame sensor available commercially off the shelf (COTS) from Hamamatsu. It has a very low background count, which was measured at 2.2 × 10^−3^ cps in laboratory lighting conditions at the National Physical Laboratory (NPL) in Teddington, UK [5]. The UVTRON responds to the incidence of photons within the 185–260 nm wavelength range on its Ni cathode. See Figure 1.

**Figure 2:**
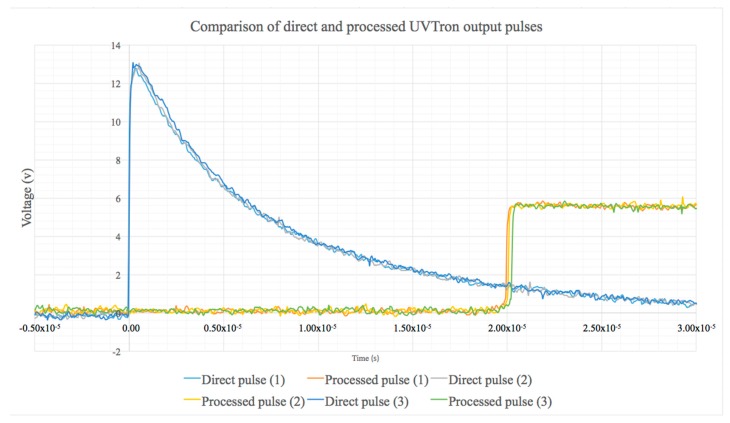
Comparison of the direct pulse recorded directly from the UVTRON and the processed pulse generated by the driver circuit (C10807, Hamamatsu) during normal UVC detection operation. The two pulses generated for three separate photon events (1, 2, 3) are shown.

Using the photoelectric effect, the sensor’s Ni cathode, a material which is only sensitive to light in the 185–260 nm wavelength range [7], emits an electron when such a photon is incident upon it. This electron is accelerated through a high-potential field towards the anode. During its transit, through the gas multiplication effect, gas within the UVTRON glass enclosure is ionised by the electron, causing the emission of further electrons, creating a cascade effect at the anode. This generates a current pulse which is emitted by the UVTRON. This can be detected directly using an oscilloscope or it can be processed by a COTS available optimised driving circuit (C10807, Hamamatsu, Japan), which both provides the high voltage required by the UVTRON and processes the output from the UVTRON into a 5 V square pulse. Figure 2 shows the direct and processed 5 V square pulse shapes from the UVTRON during normal operation.

**Figure 3:**
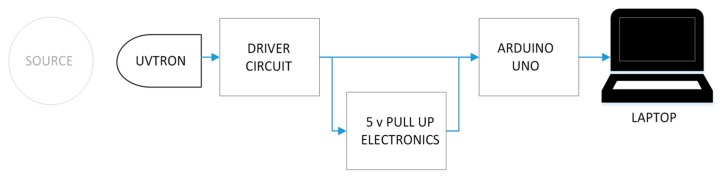
Block diagram of the set up used in these experiments.

In these experiments, the driver circuit was connected to an Arduino Uno, which counted the output pulses in each second and transmitted this to a laptop. Figure 3 shows a block diagram schematic of the set up.

This configuration of UVTRON and the driver circuit can produce a pulse approximately every 25 ms due to the quenching time of the optimised driver circuit supplied by Hamamatsu (C10807) [9]. It can therefore generate a theoretical maximum pulse rate of 40 pulses per second. In practice, the UVTRON saturates at between 35 and 37 counts per second (cps). Hamamatsu also supply a schematic of their suggested driver circuit as an alternative to their optimised, ready-made driver circuits. Used with this, the UVTRON has a minimum quenching time of 1 ms, depending on the values of the resistor and capacitor used, following the formula

tq ≈ 0.5 × C_1_·R_1_ (1)

where C_1_ is the value of the capacitor, R_1_ is the value of the resistor. Over the duration of these experiments, the UVTRON was working well below saturation levels.

The sources used at NNL were especially prepared samples of varying activity ^241^Am (100, 200, 500, 750 kBq, and 1 MBq) and a 1 MBq mixed plutonium isotope replicating what may be found in the field. A sample of ^90^Sr with an activity of approximately 11.5 kBq was also available. The sources used at Lancaster University were all sealed sources of various beta-, gamma- and alpha-emitting radionuclides with differing activities, in order that a range of emission effects could be investigated. Five different radionuclides were used in the experiments, see Table 1. Due to the low activity of the primary beta emitter, five point sources were used together to give a better level of statistical uncertainty.

**Figure 4:**
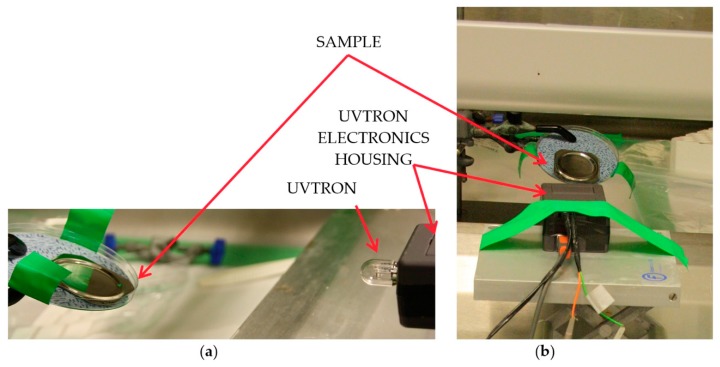
Showing the set up at the National Nuclear Laboratory (**a**) view from the side showing the vertical alignment of the UVTRON and source, and (**b**) showing the horizontal alignment of the UVTRON to the source.

For the duration of the experiments carried out at NNL, each sample was placed within a fume cupboard, and was a set distance of 170 mm from the UVTRON sensor. The UVTRON sensor and associated electronics were placed outside of the fume cupboard, but the door remained open for the duration of the experiments, see Figure 4.

The background count was taken, and comparisons were made between the different lighting options available, including the main lighting and the fume cupboard lighting. As there was no difference between any of the lighting conditions, the lights in the laboratory remained on for the duration of all experiments. Foil was inserted between the sensor and source for some of the experiments to assess the impact on the sensor count.

**Figure 5:**
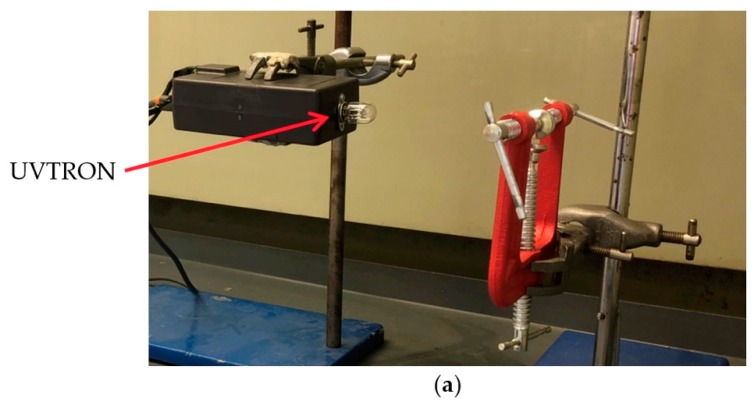

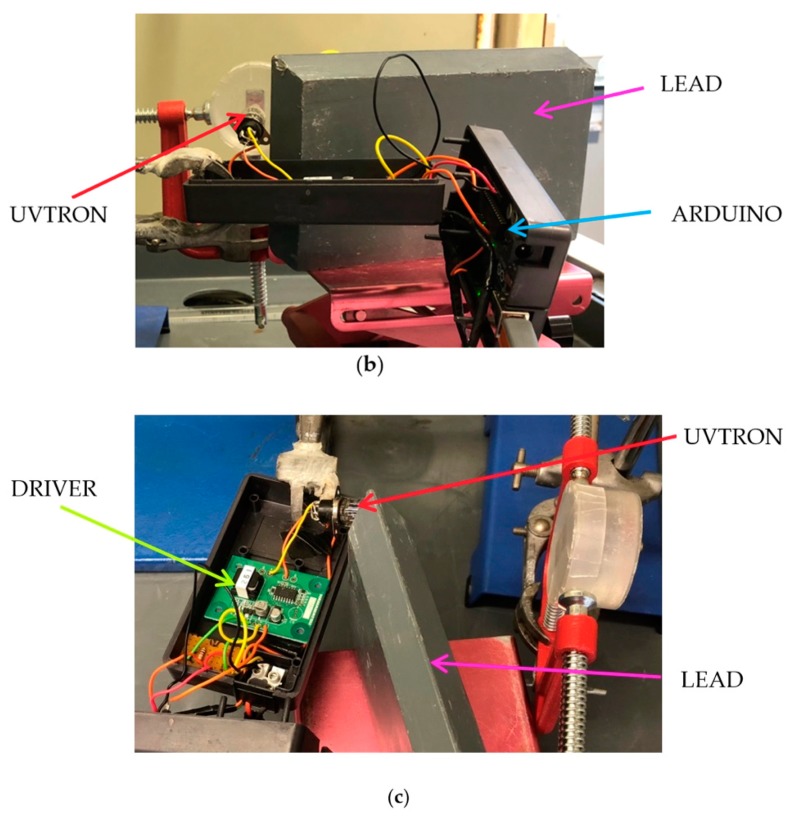
The set up at Lancaster University. (**a**) UVTRON connected to the (closed) electronics housing containing the driver circuit and Arduino microprocessor, and the clamp used to support the source. (**b**) The UVTRON is exposed to the source with lead shielding of the electronic driver circuit and Arduino microprocessor, which can just be see seen in the lid of the electronics housing. (**c**) Top view of the shielded electronics and exposed UVTRON, showing the driver circuit (green), which can be seen in the base of the electronics housing.

For the experiments carried out at Lancaster University, the source was placed at a measured distance (between 20 and 100 mm) from the cathode of the UVTRON. To gain insight into the cause of any effect on the UVTRON, several materials were inserted between the source and sensor for some of the experiments, including paper, aluminium foil, aluminium sheet of thickness 6.92 mm and lead blocks of thickness 25 mm. These were placed in close proximity to the UVTRON, see Figure 5. The number of output pulses from the UVTRON driver circuit for each second of each experiment were counted using the Arduino Uno and recorded on a laptop. These were then used to provide a gross average cps. The background response of the UVTRON in situ without the presence of any source was recorded, and this was subtracted from the count to provide a net average cps for each radionuclide, distance or inserted material. The background with the presence of the inserted materials was also recorded to determine if their presence affected the background reading; however, these were not significantly different.

## 3. Results

Where possible, the results reported here are above the critical limit as devised by Hurtgen et al. for detectors with low background counts [10]. The majority of the results are also above the limit of detection, including all ^241^Am results, with a 95.45% confidence level. Where the results differ from this, it is stated in the text. Due to the low count values, experiment durations were necessarily long, between one and three hours depending on the sample and distance, and in some instances it was necessary to rely on exceeding the critical limit to show a signal was present, though the exact magnitude of the signal could not be statistically verified.

The background was used to provide a net signal and also to identify the difference between backgrounds of different locations. The background already established at the National Physical Laboratory in previous UVTRON experiments [5] was compared with backgrounds taken at NNL and Lancaster University, see Table 2. The Lancaster University background reading is in line with the readings taken at NPL. The NNL readings were taken with the sensor facing into the fume cupboard and facing away. The background is much higher when facing the fume cupboard, which suggests that the samples housed in the fume cupboard were causing the UVTRON to respond. That this value dropped so significantly when the sensor unit was turned away would suggest that it was UVC, beta or low-energy gamma rays that could not reach or penetrate the electronics housing that was causing a reaction from the UVTRON. The background readings in both sets of experiments were taken into account in calculating a net response.

**Figure 6:**
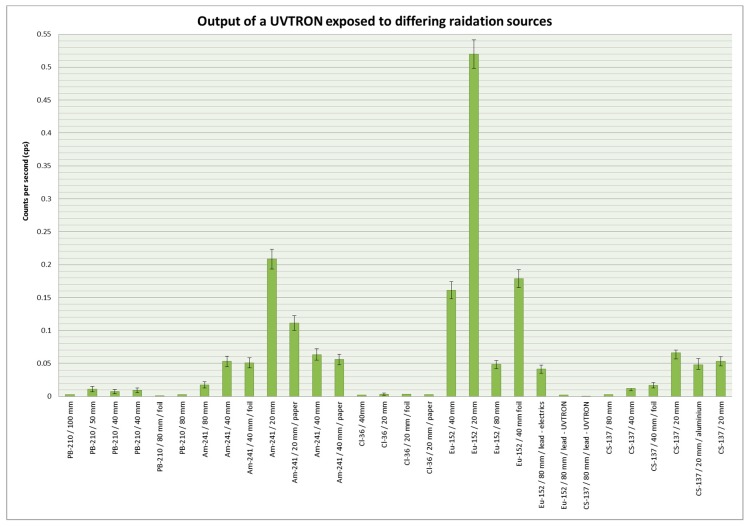
Results of the experiments carried out at Lancaster University. The error bars shown are the confidence interval as included in Table 3. Where no error bar is shown, this is due to the count being below the threshold to calculate the confidence interval, i.e., less than the limit of detection as laid out by Hurtgen et al. [10].

Results from the NNL experiments indicated that the UVTRON was susceptible to both gamma and beta radiation, though this could not be quantified at that time due to the high background readings. Hence, further experiments were planned and carried out at Lancaster University to verify the NNL findings. Figure 6 shows the average counts per second of each of the experiments. Table 3 shows the results in tabulated form.

Of the five radioisotopes tested at Lancaster University, when adjusted for the different activity levels, ^36^Cl showed the greatest count rate, with ^210^Pb second highest, ^152^Eu third, ^241^Am fourth, and ^137^Cs gave the lowest count. Assuming an isotropic distribution with a 1/r^2^ drop off, all isotopes had a count rate of less than 2% of the anticipated number of impacts onto the sensor cathode. The anticipated number of impacts was calculated using the activity of the source, the distance to the detector and the cathode size by

S_1_ = S_o_A_d_/4πr^2^, (2)

where S_1_ is the anticipated number of impacts on the sensor cathode per second, S_0_ is the activity of the source in Bq, A_d_ is the area of the sensor cathode in mm^2^, and r is the distance between the sensor and the source in mm.

The percentage of actual count relative to anticipated impacts (S_1_) was found to decline by isotope in approximately the same order as the count rate per Bq declines. ^210^Pb and ^36^Cl showed both the most counts relative to activity and the greatest percentage of expected counts due to likely impacts. ^137^Cs exhibiting the lowest count per Bq and the lowest percentage actual count relative to anticipated impacts.

When a double thickness sheet of domestic aluminium foil (thickness approx. 0.016 mm per sheet) was placed between the sensor and source, the net count for ^36^Cl, ^241^Am and ^210^Pb dropped slightly. A drop was also recorded at NNL for the ^241^Am and plutonium samples. The net count for ^152^Eu and ^137^Cs increased slightly. Using Hurtgen et al.’s method for determining uncertainty [10], there is some overlap in the reporting ranges within which the true value of the count lies. This overlap is small, and may be due to the limited duration of each of the experiments which determines the spread of the confidence interval.

When a sheet of ordinary printer paper was placed between the sensor and source the count rate for ^36^Cl and ^241^Am decreased, with no overlap in the confidence intervals for either isotope. These were the only two isotopes where paper was used to test if UVC from alpha or beta emissions were being detected. For ^36^Cl the count with paper between the source and detector was less than the count with foil between the detector and source. The drop in signal from the ^241^Am may have been due to some UVC detection, as the alpha emissions would cause radioluminescence and therefore UVC photo emission.

When a 6.92 mm thickness aluminium sheet was placed between the sensor and source the count rate for ^152^Eu and ^137^Cs dropped. The aluminium sheet was used to block beta while the higher activity gamma would be able to penetrate. These two isotopes had the highest gamma energy of those tested. There was no overlap in the confidence intervals for ^152^Eu, and a very small overlap for ^137^Cs.

Lead blocks were used to attenuate the gamma and beta incident on the sensor and electronics (see Figure 2). The UVTRON was removed from its housing and placed behind a shield, and the driver circuit and Arduino were exposed to the source. This was repeated with the electronics behind the lead shield and the UVTRON exposed to the source. The lead blocks were then used to shield the entire sensor and electronics set up. When all of the sensor and electronics were shielded the signal dropped to approximately 9% of the cps without the shielding. When the UVTRON only was shielded, the signal dropped to 4% of the cps without shielding. When the electronics were shielded and the UVTRON exposed to the source, the signal was approximately 85% of the cps without shielding. Due to the large decrease seen in signal, the limit of detection was not reached for the shielded electronics, but the critical limit was. The time to reach the limit of detection for this due to the low signal was impractical. However, the large difference in signal, although not fully quantifiable, clearly demonstrates the difference made by the location of the shielding.

## 4. Conclusions and Discussion

The results of these experiments show two disadvantages to the UVTRON as an alpha-induced radioluminescence sensor, firstly the low and variable count and secondly the susceptibility to radiation. However, now identified, the second can be overcome with correct implementation and the first taken into account in the deployment of any system using a UVTRON for alpha detection.

Due to the low count rate for relatively long durations, resulting in fractions of average counts per second, coupled with the sensitivity of the UVTRON to small changes in set up, for example, in orientation and distance, there can be significant variations on average counts per second. In addition to relatively long durations to exceed the limit of detection, and overlapping confidence intervals, it is not possible to determine absolute figures for the response of the UVTRON to radiological stimuli. However, the suggested implementation of this sensor is in an alpha detection system, where a positive or negative response to the presence of alpha radiation is required. It has been shown that this can be achieved, and nothing in these results would preclude the ability of the UVTRON to detect and locate a source if implemented correctly. As the UVTRON is designed to give an on/off response to flames, and is a sensor rather than a meter, this is also sufficient for the purpose of alpha-induced radioluminescence detection.

The UVTRON has proven to be sensitive to gamma at least in the range 47 to 344 keV and beta from 63 to 710 keV, the ranges of the main emission types and energies of the radioisotopes used in this work [11]. ^36^Cl gave the highest count per Bq, showing that the UVTRON is strongly affected by beta, which verified findings from a brief exposure to ^90^Sr at NNL. The results of using aluminium shielding indicates that gamma also affects the UVTRON as this would block beta radiation. This can also be verified by the results from the ^241^Am, which gave a significant reading with paper between the source and sensor, which would prevent any alpha and UVC photons from reaching the UVTRON, but not gamma.

So it can be seen that both gamma and beta, when incident on the UVTRON, cause an output pulse. As the Ni electrodes are not likely to be affected by the gamma or beta radiation, it may be the direct ionisation of the gas within the sensor that leads to the output pulse. Both beta and gamma would be expected to pass through the outer UV glass casing of the sensor and into the ionising gas inside, and as both are types of ionising radiation, this may initiate the cascade of electrons necessary for an output pulse from the UVTRON.

The use of lead shielding has shown that it is the UVTRON itself, rather than the associated electronics, that are primarily affected by gamma and beta radiation. This is important in any detector design using the UVTRON as the sensor element as it will need to be shielded from gamma and beta radiation, as will the electronics though not to the same degree. This lack of radiation tolerance is an issue, but one that has been seen and overcome in other electronic systems when designed for use in a nuclear environment. The use of shielding will be required if the UVTRON is to be used in an alpha detection system suitable for use in the field.

This work and the preceding work, [5,6], show that the UVTRON could be used to identify that a source is present and locate it using alpha-induced radioluminescence, but only if adequate shielding was provided to prevent both gamma and beta radiation impacting on the UVTRON. Some idea of the magnitude of the source activity could be inferred from the count rate, but the type of radionuclide could not be identified from the UVTRON readings alone. As with its use as a flame detector, the UVTRON primarily has the potential to be used as an on/off stand-off alpha detector, with the low background and the ability to use the UVTRON in daylight conditions giving it an advantage over other radioluminescence detectors of this type. In applications like the long-term storage of radioactive waste, the low-cost continual monitoring of storage facilities to ensure no leakage of radioactive materials would be one example of the benefits of a detector system based on the UVTRON, as may the continual monitoring of nuclear facilities where radioactive material contamination could be a potential occurrence.

## Figures and Tables

**Table 1:** List and properties of radionuclides.

Isotope	Activity Bq	Type of Source	Emission Type
^210^Pb	645	Point	Gamma, beta (alpha < 1%)
^241^Am	44,110	Point	Alpha, gamma
^36^Cl	50	Point	Beta
^137^Cs	16,252	Point	Beta, gamma
^152^Eu	49,830	Point	Gamma, beta
5 × ^36^Cl	5 × 50 = 250	5 × point: area 52 mm^2^ approx.	Beta

**Table 2:** Background reading comparisons.

Location	Value Av. Cps	Uncertainty cps
NPL—first visit [5]	2.2 × 10^−3^	0.7 × 10^−3^
NNL—sensor turned towards fume cupboard	23.2 × 10^−3^	2.0 × 10^−3^
NNL—sensor turned away from fume cupboard	3.2 × 10^−3^	0.7 × 10^−3^
Lancaster University	2.3 × 10^−3^	0.09 × 10^−3^

**Table 3:** Results of experiments carried out at Lancaster University.

Isotope	Activity Bq	Distance	Shielding	Counts per Second (S_net_)	Confidence Interval *
^210^Pb	645	100		0.0030	<L_d_, >L_c_
		50		0.0110	+/− 0.0039
		40		0.0073	+/− 0.0033
		40		0.0093	+/− 0.0036
		80	Foil	0.0011	<L_d_, <L_c_
		80		0.0029	<L_d_, >L_c_
^241^Am	44,110	80		0.0175	+/− 0.0048
		40		0.0530	+/− 0.0079
		40	Foil	0.0507	+/− 0.0076
		20		0.2083	+/− 0.0152
		20	Paper	0.1114	+/− 0.0112
		40		0.0634	+/− 0.0085
		40	Paper	0.0560	+/− 0.0080
5 × ^36^Cl	5 × 50 = 250	40		0.0019	<L_d_, >L_c_
		20		0.0034	+/− 0.0018
		20	Foil	0.0037	<L_d_, >L_c_
		20	Paper	0.0027	<L_d_, >L_c_
^137^Cs	16,252	80	Lead—UVTRON	0.0007	<L_d_, <L_c_
		80		0.0028	<L_d_, >L_c_
		40		0.0124	+/− 0.0037
		40	Foil	0.0172	+/− 0.0042
		20		0.0660	+/− 0.0091
		20	Aluminium	0.0481	+/− 0.0074
		20		0.0531	+/− 0.0069
^152^Eu	49,830	40		0.1610	+/− 0.0128
		20		0.5200	+/− 0.0217
		80		0.0483	+/− 0.0062
		40	Foil	0.1786	+/− 0.0136
		80	Lead—electronics	0.0412	+/− 0.0061
		80	Lead—UVTRON	0.0020	<L_d_, >L_c_

* L_d_ = limit of detection, L_c_ = critical limit, S_net_—calculated using Hurtgen et al. [10].
